# Comparative Expression of Diacylglycerol Acyltransferases for Enhanced Accumulation of Punicic Acid-Enriched Triacylglycerols in *Yarrowia lipolytica*

**DOI:** 10.3390/molecules31020281

**Published:** 2026-01-13

**Authors:** Veronika Hambalko, Simona Vevericová, Jaroslav Hambalko, Vladimír Štefuca, Peter Gajdoš, Milan Čertík

**Affiliations:** Faculty of Chemical and Food Technology, Institute of Biotechnology, Slovak University of Technology, 81237 Bratislava, Slovakia; simona.vevericova@stuba.sk (S.V.); jaroslav.hambalko@axxence.sk (J.H.); vladimir.stefuca@stuba.sk (V.Š.); peter_gajdos@stuba.sk (P.G.)

**Keywords:** punicic acid, DGAT, lipid engineering, *Yarrowia lipolytica*, waste substrates

## Abstract

Punicic acid is an uncommon ω-5 conjugated fatty acid with significant biological activity, mainly found in pomegranate seed oil. Due to limited natural availability, heterologous production of punicic acid in oleaginous yeasts offers a sustainable alternative. In this study, *Yarrowia lipolytica* was engineered for punicic acid biosynthesis by expressing the *PgFADX* gene from *Punica granatum* and subsequently modified to evaluate the influence of distinct diacylglycerol acyltransferases on punicic acid accumulation. The effects of seven acyltransferases, originating from *P. granatum* or *Y. lipolytica*, were compared under various cultivation conditions. The PgDGAT1 enzyme demonstrated the most favorable balance between total lipid content and punicic acid accumulation. Medium containing crude glycerol as a low-cost carbon source was initially tested in flask experiments with punicic acid accumulation in yeast cells of 129 mg/L. Further optimization of crude glycerol medium and subsequent scale-up experiments confirmed the potential of crude glycerol as an effective substrate, yielding up to 147.8 mg/L of punicic acid. Overall, this work identifies key enzymatic determinants for efficient punicic acid biosynthesis and supports *Y. lipolytica* as a robust host for the sustainable production of conjugated fatty acids from waste substrates.

## 1. Introduction

There is an ongoing interest in the preparation of natural bioactive compounds that could improve people’s quality of life and have a beneficial effect on their health. One such compound is punicic acid (PuA), an ω-5 fatty acid (C18:3 *cis*-9, *trans*-11, *cis*-13), which is most abundant in pomegranate seed oil (PSO), where it accounts for approximately 64–83% of all fatty acids [1]. This conjugated polyunsaturated fatty acid is known for its antioxidant, anti-inflammatory, antidiabetic, and anticancer effects [1,2]. Today, it is known that the natural production of this fatty acid is limited [3], and, therefore, it is crucial to seek alternative ways of its production. One of the promising platforms is oleaginous yeasts, particularly *Yarrowia lipolytica*. This yeast has the potential to accumulate more than 20% of its biomass in the form of lipids [4]. *Y. lipolytica* is a non-pathogenic yeast characterized by broad substrate specificity, making it a good candidate for sustainable production of value-added compounds using various industrial or agricultural waste substrates [5,6]. The use of waste substrates in biotechnological processes can reduce the cost of raw materials and contribute to the circular economy, which is beneficial from both an environmental and economic perspective [6,7,8].

Based on insights into PuA biosynthesis in pomegranate (*Punica granatum*) [3,9], this biosynthetic pathway has also been successfully introduced into *Y. lipolytica*. The basic prerequisite for its production is the expression of the *FADX* gene, which encodes a fatty acid conjugase directly responsible for the conversion of linoleic acid (LA) to PuA [9,10]. Considering that in PSO the PuA is predominantly bound in the structure of triacylglycerols (TAG), it is necessary to focus on optimizing the metabolic pathways of *Y. lipolytica* responsible for directing PuA into these structures [2]. In *Y. lipolytica*, the last step of TAG synthesis can occur through two main mechanisms: (1) the acyl-CoA-dependent pathway, which is catalyzed by the enzyme acyl-CoA:diacylglycerol acyltransferase (DGAT), and (2) the acyl-CoA-independent pathway, which is catalyzed by the enzyme phospholipid:diacylglycerol acyltransferase (PDAT). These reactions are connected to the so-called Kennedy pathway, where *sn*-glycerol-3-phosphate is gradually converted via two acylation steps to *sn*-1,2-diacylglycerol (DAG), which is then converted to TAG by DGAT. Consequently, DGAT activity has a significant impact on the level of storage lipid accumulation [11,12]. *Y. lipolytica* possesses two DGAT proteins—DGAT2 (YlDGA1) localized on the surface of lipid bodies and DGAT1 (YlDGA2) localized in a structure similar to endoplasmic reticulum. It has been proven that the lack of both DGAT proteins, together with the PDAT protein YlLRO1 in *Y. lipolytica*, led to a non-oleaginous phenotype. Interestingly, overexpression of either the *YlDGA1* or *YlDGA2* gene led to increased TAG accumulation [13,14]. Although the DGAT1 and DGAT2 enzymes are functionally related, they share no sequence homology. The *DGAT1* gene in higher plants and animals typically contains 16–17 exons compared to *DGAT2*, which consists of 8–9 exons [15,16]. DGAT1 enzymes are approximately 20 kDa larger, have more transmembrane domains than DGAT2, and their roles are thought to be specific to different organisms. There are 41 and 16 completely conserved amino acid residues, mostly located at the carboxyl termini of DGAT1 and DGAT2, respectively [17]. These enzymes also differ in substrate preference. DGAT2 is generally more active at lower acyl-CoA concentrations (≤50 µM) and less active at magnesium concentrations below 50 mM. Recent studies have also identified a third DGAT isoform, DGAT3, in *Vernicia fordii*, *Rhodotorula glutinis*, *Arabidopsis thaliana*, *Euonymus alatus*, and *Arachis hypogaea*. Unlike DGAT1 and DGAT2, DGAT3 is a soluble form of DGAT and does not contain any of the conserved residues characteristic of DGAT1 or DGAT2 [18,19,20,21].

Several studies have shown that plant DGAT isoforms may have different roles. For example, DGAT1 prefers saturated acyl groups or very long-chain acyl groups over polyunsaturated ones and plays a key role in determining the total oil content in seeds of *Arabidopsis thaliana*. Conversely, DGAT2 prefers unsaturated acyl groups and is responsible for TAGs containing specific fatty acids in oilseeds [4,22,23]. DGAT2 from *Jatropha curcas* displays a markedly higher preference for LA compared with DGAT1 in both yeast and tobacco expression systems [24]. DGAT2 from the tung tree (*V. fordii*) demonstrates a moderate substrate preference for eleostearic acid [25]. DGAT3 preferentially utilizes polyunsaturated acyl groups, and in some plants, such as *Paeonia rockii*, it shows higher expression levels than DGAT2, indicating its important role in TAG formation [22,23]. Furthermore, multiple isoforms of *DGAT* genes have been identified in many plants, e.g., four in *Physaria fendleri*, five in *P. rockii*, and up to seven in maize and rice. DGAT isoforms in *P. rockii* retain conserved motifs but have undergone parallel evolution and exhibit different physicochemical properties [23].

In addition to DGAT enzymes, the PDAT enzyme was also studied. PDAT uses an acyl group placed in the *sn*-2 position of phosphatidylcholine and phosphatidylethanolamine as an acyl group donor for DAG conversion into TAG. The *sn*-2 position of phospholipids is the place where unsaturated fatty acids such as PuA are formed. It is known that there is diversity between both DGAT homologues and PDAT from different plant species, suggesting that the substrate specificity of these enzymes is also different. An example is PDAT from *Linum usitatissimum*, which showed an increased content of α-linolenic acid in *Arabidopsis*, while native PDAT did not have such an effect. Also, there is an indication that PDAT has a major acyltransferase activity in oleaginous plants producing highly unsaturated or unusual fatty acids [12,15,26]. Comparison of DGAT/PDAT activities in related plant species, specifically sunflower and safflower seeds, demonstrated that PDAT/DGAT activity ratios were up to 19–54 times higher in safflower than in sunflower under the same conditions, depending on developmental stage and types of acyl-CoA [27].

These findings underscore the importance of selecting and testing different DGAT/PDAT isoforms for their heterologous expression in *Y. lipolytica*. Therefore, this study systematically evaluated DGAT and PDAT enzymatic activities in *Y. lipolytica* to determine their impact on PuA production under various conditions. Selected strains were further analyzed for growth and PuA accumulation using waste carbon sources as a potential inexpensive substrate. Finally, we aimed to optimize the cultivation parameters for the best-performing strain YL129 to enable scale-up from shake-flask experiments to bioreactor cultivation.

## 2. Results and Discussion

### 2.1. Overexpression of PgFADX Gene in Yarrowia lipolytica Po1d Derivative Strain

The first step in this study was to ensure the biosynthesis of PuA in *Y. lipolytica*. The *PgFADX* gene, encoding a fatty acid conjugase from *P. granatum*, was expressed in the Po1d strain under the control of the constitutive pTEF1 promoter and its more potent hybrid version, 8UAS-pTEF1. The use of a hybrid promoter has been shown by a previous study to be advantageous for the accumulation of non-traditional fatty acids in *Y. lipolytica* [28]. The resulting strain YL43 demonstrated a comparable yield of total biomass (9.7 ± 0.2 g/L) as the wild-type strain W29 (10.1 ± 0.2 g/L). Interestingly, the accumulation of total fatty acids (TFAs) in biomass was higher in the strain YL43, specifically 12.7 ± 0.3%, compared to 9.3 ± 0.2% in W29. These results suggest that the introduction of the *PgFADX* gene and subsequent PuA biosynthesis did not have a negative impact on cell growth and carbon substrate conversion into lipids.

**Figure 1:**
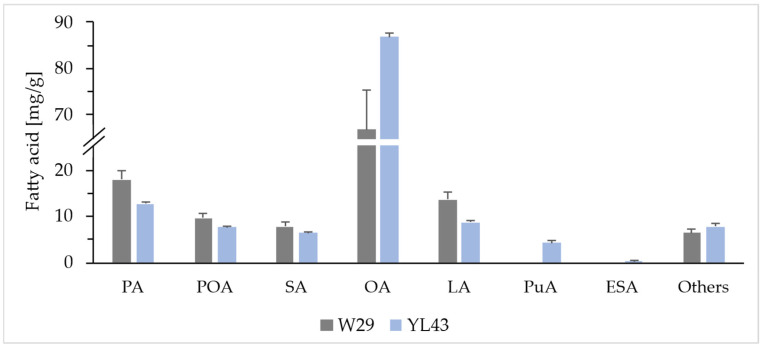
Comparison of the fatty acid profile of the YL43 strain expressing the heterologous *PgFADX* gene from *Punica granatum* with the wild-type strain W29. Both strains were cultured for 72 h in MedGL medium under the same conditions. Abbreviations: palmitic acid (PA), palmitoleic acid (POA), stearic acid (SA), oleic acid (OA), linoleic acid (LA), punicic acid (PuA), and α-eleostearic acid (ESA).

The fatty acid profile of the YL43 strain (Figure 1) indicates slight changes in the profiles of several fatty acids compared to the wild type, but the most observable effect was an increase in the oleic acid (OA) content. Conversely, the yield of LA, which serves as a direct precursor of PuA, decreased, which can be interpreted as a result of the enzymatic conversion of LA to PuA, yielding in the strain YL43 4.3 ± 0.4 mg/g of dry cell weight (DCW). In addition to the main goal, i.e., the production of PuA, the YL43 strain was also capable of producing a small amount of α-eleostearic acid (0.4 ± 0.0 mg/g DCW; ESA), which also occurs naturally in PSO [29] and whose presence in the samples was therefore caused by the activity of the FADX enzyme.

### 2.2. Monitoring the Effect of the Expression of Different Diacylglycerol Acyltransferase Genes on Punicic Acid Accumulation

The objective of this part of the work was to optimize PuA accumulation in the yeast cells by selecting a suitable DGAT/PDAT, which has the potential for high TAG productivity as well as a positive effect on PuA accumulation. Given that PuA biosynthesis is localized at the *sn*-2 position of phosphatidylcholine and for its effective accumulation in the cell, it has to be incorporated into the structure of TAGs; it is important to select suitable enzymes that are capable of effectively transferring PuA into TAGs without negatively affecting other metabolic pathways. Seven different acyl-CoA-dependent DGATs and acyl-CoA-independent PDATs were selected. Four of them, namely PgDGAT1, PgDGAT2, PgDGAT3, and PgPDAT, were derived from the plant *P. granatum*. Therefore, it was assumed that these enzymes could exhibit high specificity towards PuA as the most abundant fatty acid in PSO. The effect of these DGATs and PDAT on PuA accumulation in *Y. lipolytica* was compared with the native *Y. lipolytica*’s enzymes, namely YlDGA1, YlDGA2, and YlLRO1.

Although previous studies have tested individual acyltransferases in various heterologous hosts, for example, PgDGAT2 in *A. thaliana* [30] and *Rhodosporidium toruloides* [4], or PgPDAT in *S. cerevisiae* [31] and together with YlLRO1 in *Y. lipolytica* [10], this study represents, to our knowledge, the first comprehensive comparison of DGATs and PDAT from *P. granatum* and *Y. lipolytica* in relation to PuA accumulation. Such direct evaluation provides a perspective on the role of enzyme origin and substrate preference in tailoring lipid biosynthesis pathways for the production of conjugated fatty acids.

To determine the individual influence of selected DGATS and PDATs on the resulting accumulation of PuA, it was necessary to ensure that only one of the enzymes was expressed in the yeast. The Po1d strain, which was selected for PuA production at the outset, naturally expresses all native DGATs and PDAT and was therefore unsuitable for this experiment. The Po1d strain was replaced for this purpose by the strain Q4, in whose genome all acyltransferases responsible for the biosynthesis of storage lipids were deleted (Δ*dga1*, Δ*dga2*, Δ*lro1*, and Δ*are1*) [12]. Initially, the *PgFADX* gene under the control of the 8UAS-pTEF1 promoter was expressed in the strain Q4 (JMY1877) and already existing strains Q4-LRO1 (JMY1882), Q4-DGA2 (JMY1884), and Q4-DGA1 (JMY1892) [12], in which *YlDGA1*, *YlDGA2*, and *YlLRO1* genes were expressed under the control of the pTEF1 promoter. The resulting strains were named YL94, YL97, YL99, and YL102, respectively. Subsequently, plant *PDAT* and three *DGAT* genes were expressed in YL94, also under the control of the pTEF1 promoter, for the purpose of intercomparison and further analysis.

**Figure 2:**
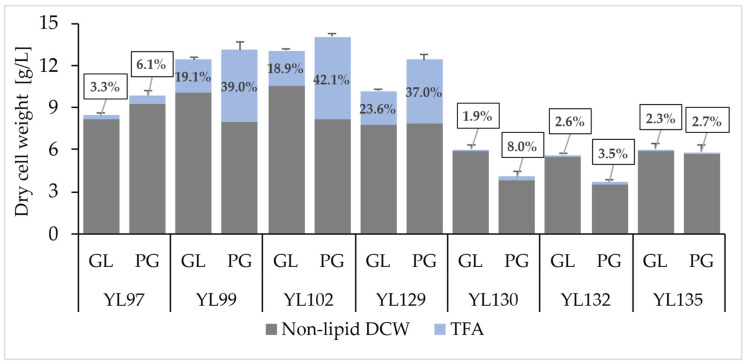
Biomass concentration expressed as the sum of the non-lipid fraction of dry cell weight (DCW) and total fatty acids (TFAs) after 72 h of cultivation of strains YL97 (*YlLRO1*), YL99 (*YlDGA2*), YL102 (*YlDGA1*), YL129 (*PgDGAT1*), YL130 (*PgDGAT2*), YL132 (*PgDGAT3*), and YL135 (*PgPDAT*) in glucose (GL) medium MedGL or pure glycerol (PG) medium MedPG.

Constructed strains were grown on glucose medium (MedGL) and pure glycerol medium (MedPG) using these compounds as the sole carbon source to compare their growth rate, TFA content in biomass, fatty acid composition, and their yields. Although slight differences in growth rate were observed among the strains, all were able to grow on MedGL and MedPG medium (Figure 2), suggesting that the expression of individual acyltransferases was not detrimental to the cells. However, considerable differences were observed in the TFA accumulation in cells, indicating that particular acyltransferases fundamentally influence the overall lipid metabolism. While strains YL97, YL130, YL132, and YL135 achieved relatively low TFA levels ranging from approximately 2–8% of DCW, significantly higher TFA values were observed in strains YL99, YL102, and YL129, where TFA content ranged from 19 to 42% of biomass. In all cases, the TFA yield was higher when cultivated on glycerol, a favorable substrate for *Y. lipolytica* [32]. These results, together with the growth rate analysis, suggest that enzymes YlDGA2, YlDGA1, and PgDGAT1 are highly efficient. Fatty acids were actively incorporated into TAG structures, allowing them to accumulate in larger quantities, and thus these enzymes represent promising candidates to synthesize TAGs rich in PuA.

The latter analysis of fatty acid profiles of examined strains revealed that, in general, almost all strains showed a higher yield of PuA when cultivated on pure glycerol. The highest absolute yield of PuA, accounting for 5.1 mg/g DCW, was observed in the strain YL130, as shown in Table 1. However, its TFA content was only 8.0% of DCW, which is considerably lower than for other strains, which achieved TFA levels of up to 42.1%. This indicates a possible preference of PgDGAT2 for incorporating PuA into TAGs, but at the same time, a low overall capacity to synthesize storage lipids in *Y. lipolytica*. The second-highest yield was observed in YL129 (4.4 mg/g DCW) when cultivated on glucose, and, therefore, enzymes of plant origin proved to be more suitable for the accumulation of PuA in *Y. lipolytica*. Even though the YL102, expressing *YlDGA1* gene, achieved the highest TFA yield on pure glycerol, its PuA accumulation was only 3.2 mg/g DCW, implying that this enzyme promotes TAG synthesis in general but is not significantly substrate-specific for PuA.

### 2.3. Punicic Acid Production on Waste Carbon Sources

**Figure 3:**
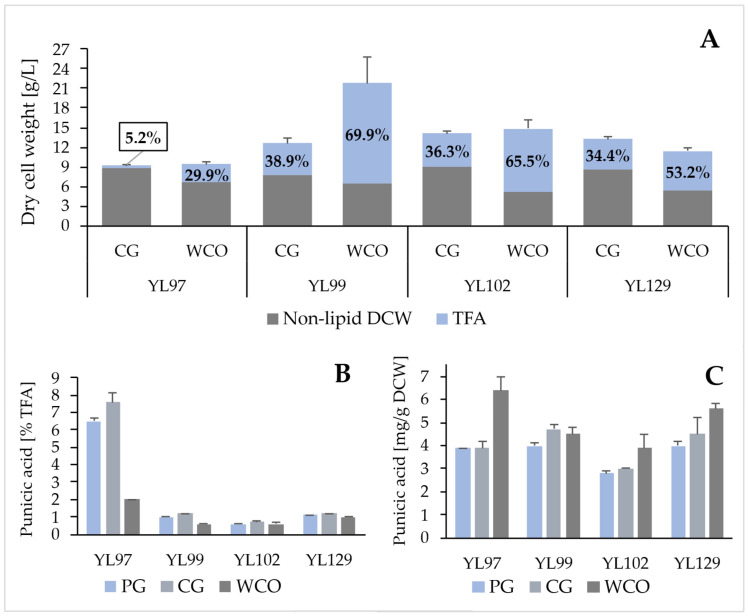
Biomass concentration expressed as the sum of the non-lipid fraction of dry cell weight (DCW) and total fatty acids (TFAs) after 72 h of cultivation of strains YL97, YL99, YL102, YL129, YL130, YL132, and YL135 in crude glycerol (CG) medium, MedCG, or waste cooking oil (WCO) medium, MedWCO (**A**), and punicic acid content in the aforementioned strains, as a percentage of TFAs (**B**) or as a yield in mg/g DCW (**C**). Data from the cultivation of these strains on pure glycerol (PG) are shown repeatedly for better visual comparison with other data presented in the figure.

*Y. lipolytica* is a yeast well known for its ability to utilize a wide range of substrates, both hydrophilic and hydrophobic [33,34,35]. Strains that showed promising results on glucose and pure glycerol were subsequently cultivated on waste substrates, namely crude glycerol and waste cooking oil (WCO). In terms of biomass growth and TFA accumulation (Figure 3A), the strains behaved almost identically in both glycerol media. In the case of WCO, a significant increase in DCW was observed in most strains, but it was found that most of the biomass was composed of lipids. The cells accumulated oil from the environment while fatty acid neosynthesis was low. This was partly confirmed by the reversed ratio of palmitoleic acid (POA) and its Δ7 isomer, which is formed by the degradation of OA. In the case of cultivation in crude glycerol medium, the POA:Δ7 ratio ranged for individual strains from 6 to 220:1, and in WCO medium, 1:10–36. The yeast received all the fatty acids necessary for the construction of its membranes from WCO, including LA, in a ratio that was natural to them.

The lowest DCW yield was observed in YL97 when growing on all four types of media. Although the native YlLRO1 acyltransferase has the unique ability to incorporate fatty acyl chains into TAG directly from the phosphatidylcholine, performed experiments indicate that its overall activity was insufficient, which was reflected in poor lipid accumulation and very low TAG accumulation in total lipids. The percentage of PuA in TFA (Figure 3B) was incomparably higher than in the other three strains, but the aforementioned negatives overshadow this fact.

YlDGA1 acyltransferase is known for its wide substrate specificity, even towards less traditional fatty acids [28]. It was therefore assumed that this acyltransferase could also be substrate-specific towards PuA, which is unusual for *Y. lipolytica*. However, the strain YL102, expressing *YlDGA1* gene, accumulated TFA in lower amounts than the strain YL99 (YlDGA2) when grown on all substrates except glucose, and PuA accumulation was also significantly lower in this strain (1.9–3.5 μg/mg DCW in YL102 depending on the carbon substrate compared to 2.5–4.7 μg/mg DCW in YL99, shown in Figure 3C). The reason for this phenomenon could be the localization of these two enzymes within the cell. It is known that YlDGA1 is located on the surface of the lipid bodies, while YlDGA2 is located on the endoplasmic reticulum [13], as is the desaturase FAD2, and it is also known that FADX conjugase is located there in plants [36]. As a result, PuA, which is synthesized on the endoplasmic reticulum, is much more accessible to YlDGA2 than to YlDGA1.

Overall, the experimental results demonstrate that, among the acyltransferases monitored above, PgDGAT1 and YlDGA2 exhibit the highest potential for efficient PuA accumulation in *Y. lipolytica*.

### 2.4. Crude Glycerol in Scale-Up of Fermentations

According to a comprehensive comparison of all monitored parameters, the YL129 strain expressing the *PgDGAT1* gene achieved the most balanced results, and it was therefore selected among the screened strains for this experiment. Despite the presence of only one DGAT enzymatic activity ensuring the accumulation of storage lipids in the form of TAGs, this strain was able to accumulate 23.6–53.2% TFA in its biomass, depending on the substrate used. At the same time, PuA production was also quite successful, reaching up to a titer of 66.4 mg/L when crude glycerol was used. It can be concluded that this carbon source is an undeniably advantageous substrate for future PuA production.

These conclusions were supported by statistical analysis. Two-way ANOVA demonstrated that both strain (F = 27.48, *p* = 5.54 × 10^−9^) and carbon source (F = 43.39, *p* = 2.20 × 10^−11^) had a highly significant effect on PuA production. In addition, there was a significant strain and carbon source interaction (F = 5.45, *p* = 15.22 × 10^−5^), indicating that the difference in PuA accumulation between strains depended on the supplied carbon substrate. Considering all monitored parameters and post hoc test results, it was found that differences in PuA production by strain YL129 compared to other strains are statistically significant in most cases. Statistically insignificant differences were observed when compared with strain YL99 during cultivation on both types of glycerol and waste cooking oil, but this does not negatively affect the selection of strain YL129 for further experiments.

#### 2.4.1. Modification of the Culture Medium and Cultivation Conditions

The first step in scaling up the process was to modify the previous medium preparation procedure so that the sterilization by filtration could be replaced by autoclaving without any precipitation of the components. The resulting combination of four separately autoclavable solutions is shown in Supplementary Table S1. As shown in Table 2, neither growth nor accumulation of TFA and PuA by the strain YL129 was affected by the sterilization method.

Regarding the composition of the culture medium, several factors were tested. The effect of using urea instead of ammonium chloride as the source of nitrogen was tested. Urea is relatively inexpensive and has a better buffering capacity than ammonium chloride, while the growth of *Y. lipolytica* in its presence is stable, and it was shown to be advantageous for lipid accumulation [37,38]. In this case, certain differences were observed (Table 2). Although the accumulation of PuA itself was positively affected, slightly lower DCW and TFA concentrations and higher residual glycerol concentrations were observed. This indicates that cell growth could be reduced by the urea used in combination with the medium sterilized by moist heat. However, since PuA accumulation was a key parameter and two-way ANOVA analysis showed that neither the change in the nitrogen source (F = 0.31, *p* = 0.60), the change in the sterilization method (F = 0.32, *p* = 0.59), nor the mutual combinations of these two factors (F = 2.08, *p* = 0.19) had a statistically significant effect on PuA titer, the medium with urea sterilized by heat was chosen for further experiments.

The concentration of ferrous cations in the environment was monitored, as well, by modifying the concentration of FeSO_4_·7H_2_O in the medium. Desaturases have a di-iron center bound by conserved histidines in their active site [39,40]. Fatty acid conjugases are homologous to FAD2-type Δ12-desaturases—they carry the same histidine motifs (HXXXXH and HXXHH) that coordinatively bind a pair of iron ions; therefore, the same cofactor (di-iron/Fe^2+^) is assumed [36,41,42]. Based on this knowledge, increasing the ferrous ion concentration could have a positive impact on PuA production. However, the results in Table 2 do not completely meet these expectations. While the PuA content in biomass increased with increasing FeSO_4_·7H_2_O concentration up to 40 mg/L, other production parameters were more or less negatively affected by this. These outcomes were also statistically analyzed in terms of the effect of changes in FeSO_4_·7H_2_O concentration. In this case, one-way ANOVA and post hoc tests were chosen. These tests proved that changes in PuA titer caused by different concentrations of ferrous cations in the medium are statistically significant (F = 5.68, *p* = 0.02), especially for a concentration of 20 mg/L, according to post hoc test results. For this reason, the original FeSO_4_·7H_2_O concentration of 20 mg/L was retained in the medium for future experiments. The final optimized medium was named MedPGU.

**Figure 4:**
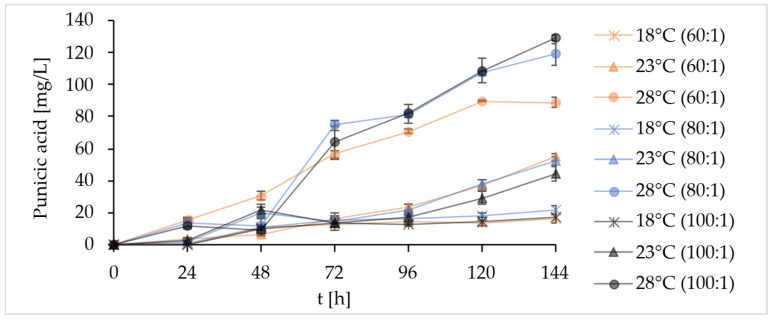
Increase in PuA titer over time during growth of the *Y. lipolytica* YL129 strain in MedPGU medium. The effect of combining three different carbon-to-nitrogen ratios (C:N = 60:1; 80:1; 100:1) and three different cultivation temperatures (18, 23, and 28 °C) was monitored.

Other factors that have a strong impact on lipid accumulation in oleaginous microorganisms are the C:N ratio [43] and temperature [44]. Lower cultivation temperatures were used since a colder environment forces cells to increase membrane fluidity by enriching their membrane with unsaturated fatty acids; therefore, lowering temperature is often a regulatory factor in desaturase induction. In parallel, the presence of molecular oxygen in the medium is necessary for desaturase activity [45]. Lower temperatures increase oxygen solubility, which could eventually have a positive effect on the accumulation of polyunsaturated fatty acids like PuA. Here, the selection of the cultivation temperature in combination with the C:N ratio optimization was performed. For this purpose, the C:N ratio was changed only by modifying the glycerol concentration. Despite the above assumptions, according to the results provided in Figure 4 and Supplementary Figure S2, a temperature of 28 °C proved to be the most favorable for PuA production. The two-way ANOVA together with the post hoc test revealed that the change in the C:N ratio itself did not have a significant effect on PuA accumulation (F = 2.77, *p* = 0.09). Conversely, the effect of temperature (F = 170.60, *p* = 1.99 × 10^−12^) or the interaction of the monitored factors (F = 3.13, *p* = 0.04) was statistically significant.

In terms of cultivation efficiency, glycerol was completely consumed only at 28 °C, regardless of the C:N ratio. At the C:N ratio of 60:1, the early glycerol depletion after 96 h was accompanied by the lowest DCW, TFA, and PuA titer (Supplementary Figure S2). Although the difference between the combination of 28 °C/C:N = 80:1 and 28 °C/C:N = 100:1 proved to be statistically insignificant, the selected strain showed a slightly higher accumulation of PuA after the specified cultivation period of 144 h when grown in a medium with a ratio of 100:1. The fatty acid profiles of strain YL129 when cultivated under the aforementioned combinations of conditions did not differ significantly from one another and are therefore not presented.

#### 2.4.2. Scale-Up of Batch Fermentations with the Strain YL129 in Bioreactor

**Figure 5:**
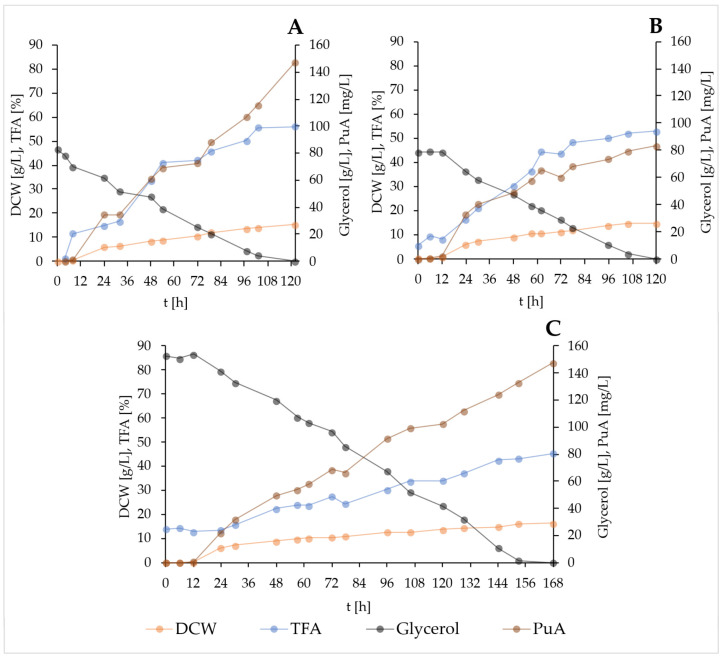
Batch fermentation in a bioreactor. Fermentation was carried out until the carbon substrate at a C:N ratio of 100:1, which was pure glycerol (80 g/L) (**A**), crude glycerol (80 g/L) (**B**), and crude glycerol (160 g/L) (**C**), was exhausted. Compared to fermentations (**A**,**B**), fermentation (**C**) had twice the concentration of nitrogen source in the medium, and the pH at the start of the process was adjusted to 5.5. Samples were taken to monitor the decrease in glycerol concentration and the increase in dry cell weight (DCW), total fatty acids (TFAs), and PuA titer.

Since glycerol is generally not an expensive substrate, especially if it originates from biodiesel production, even its use in larger quantities would not negatively affect the economic aspect of the process. For this reason, a C:N ratio of 100:1 was selected for subsequent batch fermentations. Compared to flask cultivation, summarized previously in Figure 4 and Supplementary Figure S2, carbon consumption in the bioreactor was accelerated. At the same initial glycerol concentration for the selected C:N ratio, glycerol was depleted after approximately 120 h (Figure 5A,B). Yeast cells growing in an environment with twice the amount of available carbon and nitrogen (Figure 5C) consumed glycerol slightly longer, with complete depletion observed at 168 h. Despite expectations of higher DCW in connection with higher substrate concentration, DCW peaking at 168 h (16.3 g/L) increased almost unnoticeably compared to fermentations in bioreactors with a half concentration of pure or crude glycerol (15.3 g/L and 14.6 g/L, respectively). The highest TFA accumulation during growth in flasks was 59.8% of DCW after 144 h. During fermentation in a bioreactor, it decreased by 3.7% and when using crude glycerol, by 6.9% (both after 120 h). The lowest TFA accumulation in cells (45.3%) was observed during the growth of strain YL129 in the medium with a higher initial concentration of crude glycerol. As for PuA, the results did not fully match expectations. The PuA titer in the bioreactor increased only slightly. However, a greater surprise was the almost 50% decrease in the yield of this fatty acid from 147.5 mg/L (Figure 5A) to 83.3 mg/L (Figure 5B) when the selected strain was grown in a medium containing crude glycerol.

During the first 24 h, PuA accumulated equally on both types of glycerol. During the second day of fermentation, PuA accumulation slowed down on crude glycerol, while a slight increase in polyol concentration was observed compared to pure glycerol (Supplementary Figure S3). This would explain the aforementioned decrease in the TFA quantity—part of the carbon substrate was redirected to the metabolic pathways of polyol synthesis instead of being used for lipid biosynthesis. Despite the fact that this substrate appeared to be more favorable for PuA accumulation in previous flask experiments (Figure 3), in this case, the opposite seems to be true. One of the possible explanations is that under bioreactor conditions, the impurities, such as catalyst, methanol, fatty acids, soaps, and salts [46], present in crude glycerol most likely created a stressful environment for yeast cells, resulting not only in the production of polyols but also, and more importantly, in the evident inhibition of PgFADX conjugase activity. A PuA titer of 147.8 mg/L was achieved with crude glycerol in the medium only after its concentration was doubled, and after 168 h. Extensive studies were performed to investigate PuA production across different microbial hosts (Table 3). Earlier *Y. lipolytica* studies achieved a PuA titer of 36.6 mg/L [9], and other yeasts such as *Schizosaccharomyces pombe* [47] or *S. cerevisiae* [31,41,48] generally reported lower titres compared to *Y. lipolytica*. Recent advances in oleaginous yeasts, such as *R. toruloides*, have pushed production even further, with titers of 310–451 mg/L under optimized conditions [4,49], while the most advanced fed-batch *Y. lipolytica* process reached PuA titer of grams per liter [10]. Although the PuA titer of the batch fermentation in the bioreactor in this study does not yet match these highly optimized systems, shake-flask cultures on glycerol achieved a higher titer (108.7 mg/L after 120 h, 129.1 mg/L after 144 h) compared to date highest PuA shake-flask titer observed using *Y. lipolytica*, namely 100.56 mg/L on glucose [10]. This clearly demonstrates the efficient production capacity of the strain YL129, providing a strong potential for further bioreactor optimization.

Although the increase in crude glycerol concentration did not result in an increase in PuA titer, an interesting finding of these fermentations was the relatively significant accumulation of the previously mentioned polyols, particularly mannitol and erythritol (Supplementary Figure S3). In the initial shake flask cultures using the MedPGU medium with a C:N ratio of 80:1 (Table 2), the total polyol concentration reached 14.7 g/L, of which mannitol accounted for 9.5 g/L. Among all shake flask cultures, the highest polyol accumulation was observed after changing the C:N ratio to 100:1 and temperature to 28 °C, reaching 18.0 g/L after six days, with mannitol again as the dominant metabolite (10.8 g/L). It has been proven that the maximum accumulation of polyols takes place under nitrogen-limited conditions [50], which accounts for their increased production at higher C:N ratio of 100:1. In bioreactor cultures supplemented with 80 g/L pure glycerol, the total polyol concentration increased to 21.2 g/L, with mannitol still representing the major fraction (14.1 g/L) (Supplementary Figure S3A). When crude glycerol was used, the overall concentration of polyols did not increase substantially (27.75 g/L); however, the ratio of individual polyols changed significantly. In this case, the concentrations of erythritol and mannitol were nearly equal (11.1 vs. 11.9 g/L) (Supplementary Figure S3B). At 160 g/L crude glycerol, the polyol concentration reached a maximum of 83.1 g/L observed at 129 h. High accumulation of reduced polyols (erythritol, arabitol, and mannitol) during intensive glycerol intake is a cell response to osmotic stress conditions [51]. At this stage, erythritol was the predominant polyol (51.5 g/L), followed by mannitol (22.7 g/L) and arabitol (8.9 g/L) (Supplementary Figure S3C). Several studies show that increasing the initial glycerol concentration shifts the distribution of products towards erythritol, while a lower initial concentration may favor the formation of other polyols (e.g., mannitol). Thus, “where” the carbon ends up also depends on the initial substrate concentration [52].

These findings highlight crude glycerol as a promising substrate for the combined production of PuA, accumulated in biomass, and polyols, secreted into the culture medium.

## 3. Materials and Methods

### 3.1. Culture Conditions

The bacterial strains of *Escherichia coli* were cultured in LB medium (Sigma-Aldrich, Saint-Louis, MO, USA) supplemented with the appropriate antibiotic according to the plasmid DNA composition (100 μg/mL ampicillin or 50 μg/mL kanamycin) as described by Sambrook and Russel [53].

Selection of *Yarrowia lipolytica* transformants was performed using YNBLeu [53] or YNBUra selective media. The composition of the YNBUra medium was almost identical to that of YNBLeu, but YNUra contained uracil (0.1 g/L) instead of leucine.

YPD medium (20 mL in 100 mL Erlenmeyer flasks with baffles) was used in the preparation of a 24 h yeast inoculum for the following cultivations in culture flasks. The primary medium stimulating lipid production with a C:N ratio of 80:1 used during these cultivations consisted of yeast extract (1.5 g/L), NH_4_Cl (0.5 g/L), KH_2_PO_4_ (7 g/L), Na_2_HPO_4_·12H_2_O (5 g/L), CaCl_2_ (0.1 g/L), MgSO_4_·7H_2_O (1.5 g/L), ZnSO_4_·7H_2_O (10 mg/L), FeCl_3_·6H_2_O (0.6 mg/L), MnSO_4_·H_2_O (0.07 mg/L), and CuSO_4_·5H_2_O (0.04 mg/L) and one of the following carbon sources, according to which the individual media received their designations—glucose (60 g/L; MedGL), pure glycerol (60 g/L; MedPG), crude glycerol (75 g/L—equivalent to 60 g/L pure glycerol; MedCG), or WCO (30 g/L; MedWCO). Later on, we continued the work only with the MedPG medium, the composition of which we changed in several steps. We replaced ferric chloride with ferrous sulfate in the first step. FeSO_4_·7H_2_O was tested at concentrations of 20, 30, 40, and 50 mg/L and was added from a stock III. solution, the composition of which is shown in Supplementary Table S1. Ammonium chloride was replaced by urea at a concentration of 0.28 g/L (equivalent amount of nitrogen to 0.5 g/L NH_4_Cl), and the medium was renamed the MedPGU. The previous method of media sterilization by filtration was changed to autoclave sterilization, which was possible after the MedPGU medium was divided into 4 separate solutions (Supplementary Table S1), which were mixed only after sterilization and cooling.

In general, yeast cells were grown in 50 mL of medium in 250 mL Erlenmeyer flasks with baffles for 72 h at 28 °C and 130 rpm on an orbital shaker Innova 40R (New Brunswick Scientific™, Edison, NJ, USA). In the case of monitoring yeast growth kinetics, cultivation was carried out in 100 mL of medium in 500 mL baffled flasks for 144 h, with 2 mL sampling every 24 h. During the last of the 144 h experiments, temperatures of 18, 23, and 28 °C were tested in combination with three different C:N ratios of 60:1, 80:1, and 100:1, varying only the concentration of the carbon source. The initial optical density of the medium at 600 nm (OD_600_) was 0.1 in all cases. All flask cultures were carried out in three biological replicates.

Batch cultivations were carried out in a 3 L bioreactor BioFlo (New Brunswick Scientific™) with a working volume of 2 L and double stage 6-blade Rushton turbine. The bioreactor was inoculated with the second seed culture at an initial OD_600_ = 0.2. The first seed culture was obtained in the same way as flask culture after culturing *Y. lipolytica* in the YPD medium for 24 h, and 1 mL was subsequently used to inoculate 50 mL of the MedPGU medium in a 250 mL baffled flask. The second seed cultivation also lasted 24 h (28 °C, 130 rpm). The MedPGU medium and its modifications were also chosen for bioreactor cultivation. These modifications consisted of the replacement of pure glycerol with an equivalent amount of crude glycerol (Mikrochem, Pezinok, Slovakia, specification in Supplementary Table S2) or in using twice the amount of crude glycerol in double the amount in combination with twice the amount of nitrogen source to maintain a C:N = 100:1 ratio. The culture temperature of these controlled processes was always 28 °C. The aeration was provided by an air flow rate of 0.56 vvm and an initial agitation speed of 300 rpm. Later, when the DO concentration started to decrease, the autoregulation of the agitation was set to maintain DO at 50%. Except for the fermentation with double concentration of carbon and nitrogen sources, when the pH of the medium was adjusted to 5.5 once at the beginning, the pH was not adjusted either before or during the cultivation. Fermentations were carried out until glycerol depletion. The samples were collected in 10 mL volumes and were used for continuous monitoring of glycerol and polyols concentration, DCW, and fatty acid profile.

### 3.2. Plasmid and Strain Construction

The *E. coli* strains used or constructed in this work for plasmid DNA storage and propagation were derived from the strain DH5α and are listed in Table 4. All strains of *Y. lipolytica* are also included in this table.

The amino acid sequences of the genes manipulated in this work were obtained from the NCBI database. The fatty acid conjugase gene *PgFADX* from *P. granatum* (NP_001413648.1) and four diacylglycerol acyltransferase genes *PgDGAT1* (JQ478414.1), *PgDGAT2* (JQ513387.1), *PgDGAT3* (XM_031540815.1), and *PgPDAT* (XP_031380888.1) from *P. granatum* were codon optimized for *Y. lipolytica* and synthesized by GenScript Biotech (Piscataway, NJ, USA). The genes were inserted into the JMP62 plasmid [54] with the *LEU2* or *URA3* gene as a selection marker for yeast in combination with the pTEF1 or 8UAS-pTEF1 promoters by traditional restriction cloning techniques. Both promoters were flanked by restriction sites for the endonucleases ClaI and BamHI and, in the case of the gene of interest, BamHI and AvrII. The final plasmids were formed in ligation reactions catalyzed by T4 DNA ligase (New England Biolabs, Ipswich, MA, USA). Selection of bacterial colonies after transformation was based on the presence of the *KanR* gene in the sequence of the JMP62 plasmid, which confers resistance to kanamycin. An example of the gene map for the *PgFADX* gene is provided in Supplementary Figure S1.

The insertion cassettes for transformation of *Y. lipolytica* were obtained by cleavage of plasmids with the restriction endonuclease NotI. After transformation of the yeast by the lithium acetate method [55], the transformants were selected on the YNBLeu or YNBUra medium according to the selection marker used. In the case where a strain auxotrophic for both uracil and leucine was transformed with only one insertion cassette involving the *URA3* gene, it was subsequently transformed with the intact sequence of the *LEU2* gene (obtained by cleavage of JME1868 plasmid by SalI) to repair the damaged sequence in the genome. This was necessary for all resulting yeast strains to be prototrophic.

The presence of the desired genes was confirmed in the grown bacterial or yeast colonies by polymerase chain reaction (PCR) with primers designed for the screened gene. The sequences of the primers synthesized by Microsynth (Vienna, Austria) are listed in Supplementary Table S3. The screening reactions were performed in a T100 thermocycler (Bio-Rad, Hercules, CA, USA) in the presence of OneTaq Hot Start DNA polymerase in 2X Master Mix with Standard Buffer or Q5 High-Fidelity DNA polymerase in 2X Master Mix (both from New England Biolabs).

**Table 4:** *Escherichia coli* and *Yarrowia lipolytica* strains used and constructed in the study.

Strain	Plasmid/Genotype	Reference
***E. coli***		
JME1868	4,5 kb sequence of *LEU2* gene	[3]
EC69	pUC57 *PgFADX*	Generay Biotech
EC72	JMP62 pTEF1-*PgFADX::LEU2*ex	This study
EC78	JMP62 8UAS-pTEF1-*PgFADX::LEU2*ex	This study
EC80	JMP62 8UAS-pTEF1-*PgFADX::URA3*ex	This study
EC98	pUC57 *PgPDAT*	GenScript Biotech
EC100	pUC57 *PgDGAT1*	GenScript Biotech
EC102	pUC57 *PgDGAT2*	GenScript Biotech
EC104	pUC57 *PgDGAT3*	GenScript Biotech
EC138	JMP62 pTEF1-*PgDGAT1::URA3*ex	This study
EC140	JMP62 pTEF1-*PgDGAT2::URA3*ex	This study
EC141	JMP62 pTEF1*-PgDGAT3::URA3*ex	This study
EC143	JMP62 pTEF1-*PgPDAT::URA3*ex	This study
***Y. lipolytica***		
W29	MATA, *wild type*	ATCC24060
Po1d	MATA *leu2*-270 *ura3*-302 *xpr2*-322	[56]
JMY1877 (Q4)	Po1d *Δdga1 Δlro1 Δare1 Δdga2*	[12]
JMY1882	Q4 pTEF-*YlLRO1::URA3*ex	[12]
JMY1884	Q4 pTEF-*YlDGA2::URA3*ex	[12]
JMY1892	Q4 pTEF-*YlDGA1::URA3*ex	[12]
YL43	Po1d 8UAS-pTEF1-*PgFADX::URA3*ex *LEU2*	This study
YL45	Po1d pTEF1-*PgFADX::URA3*ex *LEU2*	This study
YL94	Q4 8UAS-pTEF1-*PgFADX::LEU2*ex	This study
YL97	JMY1882 8UAS-pTEF1*-PgFADX::LEU2*ex	This study
YL99	JMY1884 8UAS-pTEF1*-PgFADX::LEU2*ex	This study
YL102	JMY1892 8UAS-pTEF1*-PgFADX::LEU2*ex	This study
YL129	YL94 pTEF1*-PgDGAT1::URA3*ex	This study
YL130	YL94 pTEF1*-PgDGAT2::URA3*ex	This study
YL132	YL94 pTEF1*-PgDGAT3::URA3*ex	This study
YL135	YL94 pTEF1*-PgPDAT::URA3*ex	This study

### 3.3. Analytical Methods

DCW was determined gravimetrically. The cell suspension was centrifuged (2880× *g*, 5 min) and washed twice with saline and once with deionized water. In the case of cell growth in the WCO medium, cells were washed three times with saline supplemented with bovine serum albumin at a concentration of 0.5% (*w*/*v*) and once with water. In the last step, the biomass was suspended in a small amount of water and lyophilized.

Samples of freeze-dried biomass were used for fatty acid analysis. Approximately 20 mg of dry biomass was weighed into a glass tube. Ballotin was added in an aliquot volume to the volume of biomass. Subsequently, 100 μL of pure hexane, 100 μL of glyceryl tritridecanoate hexane solution as an internal standard (amount equivalent to 100 µg of tridecanoic acid), and 300 μL of 0.5 M sodium methanolate in methanol (with addition of phenolphthalein) were added to the sample. The reaction mixture was stirred on a Multi Reax multi-position vortex (Heidolph, Schwabach, Germany) for 30 min at laboratory temperature. Afterwards, 765 µL of 2% acetic acid solution was added, and the sample was stirred until it became discolored. To stabilize the fatty acid methyl esters formed, 12 µL of a 1% (*w*/*v*) butylhydroxytoluene in methanol was added. The fatty acid methyl esters were extracted with 500 μL of hexane, and the extraction was carried out for 10 min on a multi-position vortex. Then the samples were centrifuged (2990× *g*, 5 min), and the organic phase was collected. The extraction with hexane was repeated one more time; the organic phases from both extractions were merged and analyzed by GC-6890 N (Agilent Technologies, Santa Clara, CA, USA) as described by Gajdoš et al. [14].

Citric acid, glucose, glycerol, mannitol, erythritol, and arabitol in media were determined by HPLC (Agilent Technologies) equipped with an Aminex HPX87H column (Bio-Rad) coupled to a RI detector and UV detector, as described by Lazar et al. [57].

Post-culture media containing WCO were hexane-extracted to analyze the amount of residual substrate. The extraction was performed twice with 50 mL of hexane. After the addition of solvent, the sample was stirred on a magnetic stirrer for 10 min. This was followed by centrifugation and transfer of the organic phase to a separatory funnel, from which the sample was spun into a pear-shaped flask through anhydrous sodium sulfate. Hexane was removed using a rotary vacuum evaporator, and residual WCO was determined gravimetrically.

The statistical software R (version R 4.5.2) was used for the initial data analysis and selection of the appropriate type of statistical test. In all cases, R selected the ANOVA test as the appropriate test for data analysis. Afterwards, statistical analyses were performed in Microsoft Excel (Microsoft 365). One-way and two-way ANOVA were used to assess the effects of individual factors and their interactions. When ANOVA indicated significant differences (*p* < 0.05), appropriate post hoc pairwise comparisons were carried out.

## 4. Conclusions

This study successfully established *Yarrowia lipolytica* as an efficient microbial platform for the heterologous biosynthesis of PuA from *Punica granatum*. Through systematic evaluation of DGATs and PDATs originating from both yeast and plant sources, the PgDGAT1 enzyme was identified as the most promising candidate for the incorporation of PuA into TAGs while maintaining a high TFA content. The comparative analysis confirmed that enzyme origin, intracellular localization, and substrate preference strongly affect the final distribution of fatty acids within the lipid fraction.

Cultivation optimization demonstrated that medium composition, temperature, and concentration of carbon substrate are crucial for balancing growth and product yield. Among the tested conditions, a C:N ratio of 100:1 and cultivation at 28 °C provided the highest PuA titers. Furthermore, the use of crude glycerol, a by-product of biodiesel production, was shown to be an economically and environmentally attractive carbon source, supporting both PuA and polyol synthesis. Although high glycerol concentrations induced osmotic stress and redirected part of the carbon flux toward polyol synthesis, this dual production system offers potential for integrated bioprocesses combining lipid and polyol recovery.

Overall, the results contribute to a deeper understanding of lipid metabolism engineering in *Y. lipolytica* and highlight the feasibility of using renewable, low-cost substrates for the sustainable microbial production of valuable conjugated fatty acids.

## Figures and Tables

**Table 1:** Content of the most abundant and unusual fatty acids in strains YL97 (*YlLRO1*), YL99 (*YlDGA2*), YL102 (*YlDGA1*), YL129 (*PgDGAT1*), YL130 (*PgDGAT2*), YL132 (*PgDGAT3*), and YL135 (*PgPDAT*) when grown for 72 h in glucose (GL) medium, MedGL, or pure glycerol (PG) medium, MedPG. Abbreviations: palmitic acid (PA), palmitoleic acid (POA), stearic acid (SA), oleic acid (OA), linoleic acid (LA), punicic acid (PuA), α-eleostearic acid (ESA).

Strain	Carbon Source	Fatty Acid [mg/g DCW]							
		PA	POA	SA	OA	LA	PuA	ESA	Others
YL97	GL	3.5 ± 0.3	2.2 ± 0.2	0.5 ± 0.0	24.8 ± 2.0	6.7 ± 0.6	2.6 ± 0.3	0.3 ± 0.0	2.5 ± 0.3
	PG	3.6 ± 0.4	3.3 ± 0.1	1.6 ± 0.2	35.6 ± 1.3	10.5 ± 0.0	3.9 ± 0.0	0.3 ± 0.0	2.1 ± 0.2
YL99	GL	25.2 ± 2.7	10.8 ± 0.8	9.9 ± 1.0	125 ± 12	8.1 ± 0.8	2.5 ± 0.3	0.5 ± 0.1	8.8 ± 1.0
	PG	40.9 ± 1.0	25.1 ± 0.5	24.0 ± 0.6	264.0 ± 6.7	17.5 ± 0.5	4.0 ± 0.1	0.5 ± 0.0	13.8 ± 0.5
YL102	GL	14.0 ± 2.3	6.4 ± 1.0	11.9 ± 1.9	85.0 ± 13.5	7.9 ± 0.8	1.9 ± 0.2	0.4 ± 0.1	9.7 ± 1.0
	PG	52 ± 12	30.8 ± 7.2	56 ± 12	265.0 ± 6.2	19.0 ± 4.3	3.2 ± 0.6	0.5 ± 0.1	23.8 ± 5.5
YL129	GL	24.3 ± 2.7	10.6 ± 1.3	18.0 ± 0.7	151.5 ± 9.4	12.4 ± 0.9	4.4 ± 0.1	0.8 ± 0.1	13.8 ± 1.1
	PG	32.9 ± 1.4	15.1 ± 0.5	42.8 ± 2.5	242.9 ± 4.0	14.0 ± 0.1	4.0 ± 0.2	0.5 ± 0.1	18.0 ± 1.2
YL130	GL	2.6 ± 0.6	0.8 ± 0.1	0.2 ± 0.1	7.3 ± 1.6	4.4 ± 0.9	1.5 ± 0.4	0.3 ± 0.1	2.1 ± 0.5
	PG	8.9 ± 1.4	5.0 ± 0.9	2.1 ± 0.4	24.9 ± 5.1	30.0 ± 5.6	5.1 ±0.1	0.5 ± 0.0	2.3 ± 0.3
YL132	GL	3.1 ± 0.1	1.0 ± 0.0	0.2 ± 0.0	8.2 ± 0.1	5.5 ± 0.1	1.9 ± 0.1	0.3 ± 0.0	2.4 ± 0.2
	PG	5.2 ± 1.7	2.9 ± 1.0	1.0 ± 0.2	13.6 ± 4.5	16.0 ± 5.5	3.1 ± 0.8	0.3 ± 0.1	2.1 ± 0.7
YL135	GL	3.4 ± 0.0	0.9 ± 0.0	0.3 ± 0.0	8.5 ± 0.4	5.5 ± 0.2	1.9 ± 0.1	0.3 ± 0.0	2.4 ± 0.2
	PG	1.8 ± 0.9	1.0 ± 0.4	0.6 ± 0.4	8.6 ± 3.2	6.5 ± 2.4	1.7 ± 0.6	0.2 ± 0.1	1.2 ± 0.5

**Table 2:** Flask cultivation of the *Y. lipolytica* YL129 strain in MedPG medium focused on monitoring the effect of sterilization method, nitrogen source type, and ferrous cation concentration on the growth and production properties of the selected strain. Abbreviations: dry cell weight (DCW), residual glycerol (Gly_RES_), total fatty acids (TFAs), punicic acid (PuA).

	Filter Sterilization		Moist Heat Sterilization				
FeSO_4_·7H_2_O	MedPG	MedPG + Urea	MedPG	MedPG + Urea			
	20 mg/L	20 mg/L	20 mg/L	20 mg/L	30 mg/L	40 mg/L	50 mg/L
DCW [g/L]	13.1 ± 0.7	13.5 ± 0.1	13.4 ± 0.2	10.7 ± 0.1	9.4 ± 0.4	8.5 ± 1.8	6.2 ± 1.5
Gly_RES_ [g/L]	3.4 ± 1.3	6.0 ± 1.6	3.3 ± 0.7	10.4 ± 1.6	16.1 ± 1.5	16.0 ± 3.9	19.3 ± 4.0
TFA [% DCW]	43.0 ± 1.6	41.0 ± 2.4	44.2 ± 1.0	31.0 ± 2.7	33.8 ± 3.0	38.5 ± 0.5	25.7 ± 5.4
PuA [% TFA]	1.3 ± 0.7	1.9 ± 0.3	1.2 ± 0.1	2.2 ± 0.0	2.1 ± 0.0	1.9 ± 0.2	2.3 ± 0.1
PuA [mg/g DCW]	5.5 ± 0.2	6.0 ± 0.6	5.4 ± 0.5	6.7 ± 0.6	7.2 ± 0.6	7.4 ± 0.8	5.8 ± 1.0
PuA [mg/L]	71.6 ± 1.5	80.4 ± 5.7	72.2 ± 4.8	71.9 ± 6.1	67.8 ± 7.1	64.1 ± 18.0	37 ± 13

**Table 3:** Overview of previous studies on the production of punicic acid in various microorganisms. Abbreviations: punicic acid (PuA), linoleic acid (LA), and total fatty acids (TFAs).

Host Organism	Engineering Strategy	Substrate	PuA Production	Reference
*Y. lipolytica*	*PgFADX*, *YlDGA2*, *GPD1*, *eyk1Δ*, *pox1-6Δ*, *tgl4Δ*	Glucose	36.6 mg/L (shake-flask)	[9]
*Y* *. lipolytica*	*PgFADX*, *YlFAD2*, *YlCPT*, *PgLPCAT*, *YlLRO1*, *GPD1*, *P. graminis Δ9 desaturase*, *MaELO2*, *YlELO1*, *pex10Δ*, *gut2Δ*, *lro1Δ*, *fad2Δ*, *scdΔ*, *lip1Δ*, *scp2Δ*	Glucose	100.56 mg/L (shake-flask) 3072.72 mg/L (bioreactor)	[10]
*S*. *cerevisiae*	*PgFADX*	Glucose, galactose, and LA	1.6% of the TFA (shake-flask)	[48]
*S*. *cerevisiae*	*TkFADX*	Galactose and LA	0.1% of the TFA (shake-flask)	[41]
*S*. *cerevisiae*	*PgFADX*	Galactose and LA	0.8% of the TFA (shake-flask)	[41]
*S*. *cerevisiae*	*PgFADX*, *PgPDAT*, *PgLPCAT*, *snf2Δ*	LA	7.2 mg/L (shake-flask)	[31]
*S*. *pombe*	*PgFADX*	Glucose	38.71 mg/L (shake-flask)	[47]
*S. pombe*	*PgFADX*, *PgFAD2*	Glucose	34.33 mg/L (shake-flask)	[47]
*R. toruloides*	*PgFADX*	Glucose	105.77 mg/L (shake-flask)	[49]
*R. toruloides*	*PgFADX*	Crude glycerol	72.81 mg/L (shake-flask)	[49]
*R. toruloides*	*PgFADX*, *PgFAD2*	Glucose	451.6 mg/L (shake-flask)	[4]
*R. toruloides*	*PgFADX*, *PgFAD2*	Wood hydrolysate	310 mg/L (shake-flask)	[4]

## Data Availability

The data are contained within the article and Supplementary Materials.

## Supplementary Materials

The following supporting information can be downloaded at: https://www.mdpi.com/article/10.3390/molecules31020281/s1. Figure S1: Example of a plasmid gene map with the JMP62 base from *E. coli* strains EC78 and E80; Figure S2: Growth kinetics of *Y. lipolytica* YL129 strain in MedPGU; Figure S3: Polyol production of *Y. lipolytica* YL129 strain; Table S1: Composition of solutions for the preparation of MedPGU medium; Table S2: Official specifications of crude glycerol used in this study from the manufacturer; Table S3: Primers used in the study.

## Abbreviations

The following abbreviations are used in this manuscript:

DAG	Diacylglycerol
DCW	Dry cell weight
DGAT	Acyl-CoA:diacylglycerol acyltransferase
DO	Dissolved oxygen
ESA	α-eleostearic acid
FA	Fatty acid
LA	Linoleic acid
OA	Oleic acid
PA	Palmitic acid
PDAT	Phospholipid:diacylglycerol acyltransferase
POA	Palmitoleic acid
PSO	Pomegranate seed oil
PuA	Punicic acid
TAG	Triacylglycerol
TFA	Total fatty acids
WCO	Waste cooking oil

## References

[1] Almoraie M., Spencer J., Wagstaff C. (2025). Fatty Acid Profile, Tocopherol Content, and Phenolic Compounds of Pomegranate (*Punica granatum* L.) Seed Oils. J. Food Compos. Anal..

[2] Zhou D., Zhao M., Wang J., Faiza M., Chen X., Cui J., Liu N., Li D. (2022). A Novel and Efficient Method for Punicic Acid-Enriched Diacylglycerol Preparation: Enzymatic Ethanolysis of Pomegranate Seed Oil Catalyzed by Lipozyme 435. Lebensm.-Wiss. Technol..

[3] Holic R., Xu Y., Caldo K.M.P., Singer S.D., Field C.J., Weselake R.J., Chen G. (2018). Bioactivity and Biotechnological Production of Punicic Acid. Appl. Microbiol. Biotechnol..

[4] Wang J., Haddis D.Z., Xiao Q., Bressler D.C., Chen G. (2024). Engineering *Rhodosporidium Toruloides* for Sustainable Production of Value-Added Punicic Acid from Glucose and Wood Residues. Bioresour. Technol..

[5] Elsharawy H., Refat M. (2024). *Yarrowia lipolytica*: A Promising Microbial Platform for Sustainable Squalene Production. Biocatal. Agric. Biotechnol..

[6] Lopes M., Miranda S.M., Costa A.R., Pereira A.S., Belo I. (2022). *Yarrowia lipolytica* as a Biorefinery Platform for Effluents and Solid Wastes Valorization—Challenges and Opportunities. Crit. Rev. Biotechnol..

[7] Gottardi D., Siroli L., Vannini L., Patrignani F., Lanciotti R. (2021). Recovery and Valorization of Agri-Food Wastes and by-Products Using the Non-Conventional Yeast *Yarrowia lipolytica*. Trends Food Sci. Technol..

[8] Nguyen Q.D., Nguyen T.-V.-L., Tran T.T.V., Khatri Y., Chandrapala J., Truong T. (2025). Single Cell Oils from Oleaginous Yeasts and Metabolic Engineering for Potent Cultivated Lipids: A Review with Food Application Perspectives. Future Foods.

[9] Urbanikova V., Park Y.-K., Krajciova D., Tachekort M., Certik M., Grigoras I., Holic R., Nicaud J.-M., Gajdos P. (2023). *Yarrowia lipolytica* as a Platform for Punicic Acid Production. Int. J. Mol. Sci..

[10] Wang K., Zhou Y., Cao L., Lin L., Ledesma-Amaro R., Ji X.-J. (2024). Engineering *Yarrowia lipolytica* for Sustainable Production of the Pomegranate Seed Oil-Derived Punicic Acid. J. Agric. Food Chem..

[11] Xu J., Francis T., Mietkiewska E., Giblin E.M., Barton D.L., Zhang Y., Zhang M., Taylor D.C. (2008). Cloning and Characterization of an Acyl-CoA-Dependent Diacylglycerol Acyltransferase 1 (DGAT1) Gene from *Tropaeolum majus*, and a Study of the Functional Motifs of the DGAT Protein Using Site-Directed Mutagenesis to Modify Enzyme Activity and Oil Content. Plant Biotechnol. J..

[12] Beopoulos A., Haddouche R., Kabran P., Dulermo T., Chardot T., Nicaud J.-M. (2012). Identification and Characterization of DGA2, an Acyltransferase of the DGAT1 Acyl-CoA:Diacylglycerol Acyltransferase Family in the Oleaginous Yeast *Yarrowia lipolytica*. New Insights into the Storage Lipid Metabolism of Oleaginous Yeasts. Appl. Microbiol. Biotechnol..

[13] Gajdoš P., Ledesma-Amaro R., Nicaud J.-M., Čertík M., Rossignol T. (2016). Overexpression of Diacylglycerol Acyltransferase in *Yarrowia lipolytica* Affects Lipid Body Size, Number and Distribution. FEMS Yeast Res..

[14] Gajdoš P., Nicaud J.-M., Rossignol T., Čertík M. (2015). Single Cell Oil Production on Molasses by *Yarrowia lipolytica* Strains Overexpressing DGA2 in Multicopy. Appl. Microbiol. Biotechnol..

[15] Chen G., Harwood J.L., Lemieux M.J., Stone S.J., Weselake R.J. (2022). Acyl-CoA:Diacylglycerol Acyltransferase: Properties, Physiological Roles, Metabolic Engineering and Intentional Control. Prog. Lipid Res..

[16] Liu Q., Siloto R.M.P., Lehner R., Stone S.J., Weselake R.J. (2012). Acyl-CoA:Diacylglycerol Acyltransferase: Molecular Biology, Biochemistry and Biotechnology. Prog. Lipid Res..

[17] Cao H. (2018). Identification of the Major Diacylglycerol Acyltransferase mRNA in Mouse Adipocytes and Macrophages. BMC Biochem..

[18] Turchetto-Zolet A.C., Maraschin F.S., de Morais G.L., Cagliari A., Andrade C.M., Margis-Pinheiro M., Margis R. (2011). Evolutionary View of Acyl-CoA Diacylglycerol Acyltransferase (DGAT), a Key Enzyme in Neutral Lipid Biosynthesis. BMC Evol. Biol..

[19] Rani S.H., Krishna T.H.A., Saha S., Negi A.S., Rajasekharan R. (2010). Defective in Cuticular Ridges (DCR) of *Arabidopsis thaliana*, a Gene Associated with Surface Cutin Formation, Encodes a Soluble Diacylglycerol Acyltransferase. J. Biol. Chem..

[20] Cao H., Shockey J.M., Klasson K.T., Chapital D.C., Mason C.B., Scheffler B.E. (2013). Developmental Regulation of Diacylglycerol Acyltransferase Family Gene Expression in Tung Tree Tissues. PLoS ONE.

[21] Cai W.-L., Yu S.-Y., Hu Y.-H. (2025). Synergistic Mechanisms of DGAT and PDAT in Shaping Triacylglycerol Diversity: Evolutionary Insights and Metabolic Engineering Strategies. Front. Plant Sci..

[22] Yu L., Zhou C., Fan J., Shanklin J., Xu C. (2021). Mechanisms and Functions of Membrane Lipid Remodeling in Plants. Plant J..

[23] Xu H., Li M., Ma D., Gao J., Tao J., Meng J. (2024). Identification of Key Genes for Triacylglycerol Biosynthesis and Storage in Herbaceous Peony (*Paeonia lactifolra* Pall.) Seeds Based on Full-Length Transcriptome. BMC Genom..

[24] Xu R., Yang T., Wang R., Liu A. (2014). Characterisation of DGAT1 and DGAT2 from *Jatropha Curcas* and Their Functions in Storage Lipid Biosynthesis. Funct. Plant Biol. FPB.

[25] Shockey J.M., Gidda S.K., Chapital D.C., Kuan J.-C., Dhanoa P.K., Bland J.M., Rothstein S.J., Mullen R.T., Dyer J.M. (2006). Tung Tree DGAT1 and DGAT2 Have Nonredundant Functions in Triacylglycerol Biosynthesis and Are Localized to Different Subdomains of the Endoplasmic Reticulum. Plant Cell.

[26] Marmon S., Sturtevant D., Herrfurth C., Chapman K., Stymne S., Feussner I. (2017). Two Acyltransferases Contribute Differently to Linolenic Acid Levels in Seed Oil1. Plant Physiol..

[27] Banaś W., Sanchez Garcia A., Banaś A., Stymne S. (2013). Activities of Acyl-CoA:Diacylglycerol Acyltransferase (DGAT) and Phospholipid:Diacylglycerol Acyltransferase (PDAT) in Microsomal Preparations of Developing Sunflower and Safflower Seeds. Planta.

[28] Gajdoš P., Urbaníková V., Vicenová M., Čertík M. (2022). Enhancing Very Long Chain Fatty Acids Production in *Yarrowia lipolytica*. Microb. Cell Factories.

[29] Loukhmas S., Kerak E., Elgadi S., Ettalibi F., El Antari A., Harrak H. (2021). Oil Content, Fatty Acid Composition, Physicochemical Properties, and Antioxidant Activity of Seed Oils of Ten Moroccan Pomegranate Cultivars. J. Food Qual..

[30] Weselake R.J., Mietkiewska E. (2014). Gene Combinations for Producing Punicic Acid in Transgenic Plants. U.S. Patent.

[31] Wang J., Xu Y., Holic R., Yu X., Singer S.D., Chen G. (2021). Improving the Production of Punicic Acid in Baker’s Yeast by Engineering Genes in Acyl Channeling Processes and Adjusting Precursor Supply. J. Agric. Food Chem..

[32] Tsirigka A., Theodosiou E., Patsios S.I., Tsoureki A., Andreadelli A., Papa E., Aggeli A., Karabelas A.J., Makris A.M. (2023). Novel Evolved *Yarrowia lipolytica* Strains for Enhanced Growth and Lipid Content under High Concentrations of Crude Glycerol. Microb. Cell Factories.

[33] Spagnuolo M., Shabbir Hussain M., Gambill L., Blenner M. (2018). Alternative Substrate Metabolism in *Yarrowia lipolytica*. Front. Microbiol..

[34] Wierzchowska K., Derewiaka D., Zieniuk B., Nowak D., Fabiszewska A. (2023). Whey and Post-Frying Oil as Substrates in the Process of Microbial Lipids Obtaining: A Value-Added Product with Nutritional Benefits. Eur. Food Res. Technol..

[35] Wierzchowska K., Szulc K., Zieniuk B., Fabiszewska A. (2025). Bioconversion of Liquid and Solid Lipid Waste by *Yarrowia lipolytica* Yeast: A Study of Extracellular Lipase Biosynthesis and Microbial Lipid Production. Molecules.

[36] Dyer J.M., Chapital D.C., Kuan J.-C.W., Mullen R.T., Turner C., McKeon T.A., Pepperman A.B. (2002). Molecular Analysis of a Bifunctional Fatty Acid Conjugase/Desaturase from Tung. Implications for the Evolution of Plant Fatty Acid Diversity. Plant Physiol..

[37] Brabender M., Hussain M.S., Rodriguez G., Blenner M.A. (2018). Urea and Urine Are a Viable and Cost-Effective Nitrogen Source for *Yarrowia lipolytica* Biomass and Lipid Accumulation. Appl. Microbiol. Biotechnol..

[38] Konzock O., Zaghen S., Fu J., Kerkhoven E.J. (2022). Urea Is a Drop-in Nitrogen Source Alternative to Ammonium Sulphate in *Yarrowia lipolytica*. iScience.

[39] Shanklin J., Guy J.E., Mishra G., Lindqvist Y. (2009). Desaturases: Emerging Models for Understanding Functional Diversification of Diiron-Containing Enzymes. J. Biol. Chem..

[40] Lindqvist Y., Huang W., Schneider G., Shanklin J. (1996). Crystal Structure of Delta9 Stearoyl-acyl Carrier Protein Desaturase from Castor Seed and Its Relationship to Other Di-iron Proteins. EMBO J..

[41] Iwabuchi M., Kohno-Murase J., Imamura J. (2003). Δ12-Oleate Desaturase-Related Enzymes Associated with Formation of Conjugated *Trans*-Δ11, *Cis*-Δ13 Double Bonds. J. Biol. Chem..

[42] Bláhová Z., Harvey T.N., Pšenička M., Mráz J. (2020). Assessment of Fatty Acid Desaturase (Fads2) Structure-Function Properties in Fish in the Context of Environmental Adaptations and as a Target for Genetic Engineering. Biomolecules.

[43] Sestric R., Munch G., Cicek N., Sparling R., Levin D.B. (2014). Growth and Neutral Lipid Synthesis by *Yarrowia lipolytica* on Various Carbon Substrates under Nutrient-Sufficient and Nutrient-Limited Conditions. Bioresour. Technol..

[44] Tezaki S., Iwama R., Kobayashi S., Shiwa Y., Yoshikawa H., Ohta A., Horiuchi H., Fukuda R. (2017). Δ12-Fatty Acid Desaturase Is Involved in Growth at Low Temperature in Yeast *Yarrowia lipolytica*. Biochem. Biophys. Res. Commun..

[45] Kobalter S., Wriessnegger T., Pichler H. (2025). Engineering Yeast for Tailored Fatty Acid Profiles. Appl. Microbiol. Biotechnol..

[46] Zhao M., Wang Y., Zhou W., Zhou W., Gong Z. (2023). Co-Valorization of Crude Glycerol and Low-Cost Substrates via Oleaginous Yeasts to Micro-Biodiesel: Status and Outlook. Renew. Sustain. Energy Rev..

[47] Garaiova M., Mietkiewska E., Weselake R.J., Holic R. (2017). Metabolic Engineering of *Schizosaccharomyces pombe* to Produce Punicic Acid, a Conjugated Fatty Acid with Nutraceutic Properties. Appl. Microbiol. Biotechnol..

[48] Hornung E., Pernstich C., Feussner I. (2002). Formation of Conjugated Δ11Δ13-Double Bonds by Δ12-Linoleic Acid (1,4)-Acyl-Lipid-Desaturase in Pomegranate Seeds. Eur. J. Biochem..

[49] Krajciova D., Holic R. (2024). The Plasma Membrane H+-ATPase Promoter Driving the Expression of FADX Enables Highly Efficient Production of Punicic Acid in *Rhodotorula toruloides* Cultivated on Glucose and Crude Glycerol. J. Fungi Basel Switz..

[50] Papanikolaou S., Diamantopoulou P., Blanchard F., Lambrinea E., Chevalot I., Stoforos N.G., Rondags E. (2020). Physiological Characterization of a Novel Wild-Type *Yarrowia lipolytica* Strain Grown on Glycerol: Effects of Cultivation Conditions and Mode on Polyols and Citric Acid Production. Appl. Sci..

[51] Szczepańczyk M., Rzechonek D.A., Neuvéglise C., Mirończuk A.M. (2023). In-Depth Analysis of Erythrose Reductase Homologs in *Yarrowia lipolytica*. Sci. Rep..

[52] Vastaroucha E.-S., Maina S., Michou S., Kalantzi O., Pateraki C., Koutinas A.A., Papanikolaou S. (2021). Bioconversions of Biodiesel-Derived Glycerol into Sugar Alcohols by Newly Isolated Wild-Type *Yarrowia lipolytica* Strains. Reactions.

[53] Sambrook J. (2001). Molecular Cloning: A Laboratory Manual.

[54] Nicaud J.-M., Madzak C., van den Broek P., Gysler C., Duboc P., Niederberger P., Gaillardin C. (2002). Protein Expression and Secretion in the Yeast *Yarrowia lipolytica*. FEMS Yeast Res..

[55] Le Dall M.-T., Nicaud J.-M., Gaillardin C. (1994). Multiple-Copy Integration in the Yeast *Yarrowia lipolytica*. Curr. Genet..

[56] Barth G., Gaillardin C., Wolf K. (1996). *Yarrowia lipolytica*. Nonconventional Yeasts in Biotechnology: A Handbook.

[57] Lazar Z., Walczak E., Robak M. (2011). Simultaneous Production of Citric Acid and Invertase by *Yarrowia lipolytica SUC*+ Transformants. Bioresour. Technol..

