# Supplementary material for: Comparative Expression of Diacylglycerol Acyltransferases for Enhanced Accumulation of Punicic Acid-Enriched Triacylglycerols in Yarrowia lipolytica
Source: Molecules. 2026 Jan 13;31(2):281. doi: 10.3390/molecules31020281 (PMC12844487; doi:10.3390/molecules31020281)
Supplement: Supplementary file 1 [file molecules-31-00281-s001.zip › molecules-4010619-supplementary.pdf]

Supplementary information for:

Comparative Expression of Diacylglycerol Acyltransferases for Enhanced Accumulation of Punicic Acid-Enriched Triacylglycerols in *Yarrowia lipolytica*

Veronika Hambalko *, Simona Vevericová, Jaroslav Hambalko, Vladimír Štefuca, Peter Gajdoš and Milan Čertík *

Faculty of Chemical and Food Technology, Institute of Biotechnology, Slovak University of Technology, 81237 Bratislava, Slovakia; simona.vevericova@stuba.sk (S.V.); jaroslav.hambalko@axxence.sk (J.H.); vladimir.stefuca@stuba.sk (V.Š.); peter_gajdos@stuba.sk (P.G.)

* Correspondence: veronika.hambalko@stuba.sk (V.H.); milan.certik@stuba.sk (M.Č.)

Table of contents **Page**

Supplementary Figure S1. Example of a plasmid gene map with the JMP62 base from *E. coli* strains EC78 and E80. 2

Supplementary Figure S2. Growth kinetics of *Y. lipolytica* YL129 strain in MedPGU. 3

Supplementary Figure S3. Polyol production of *Y. lipolytica* YL129 strain. 4

Supplementary Table S1. Composition of solutions for the preparation of MedPGU medium. 5

Supplementary Table S2. Official specifications of crude glycerol used in this study from the manufacturer. 6

Supplementary Table S3. Primers used in the study. 7

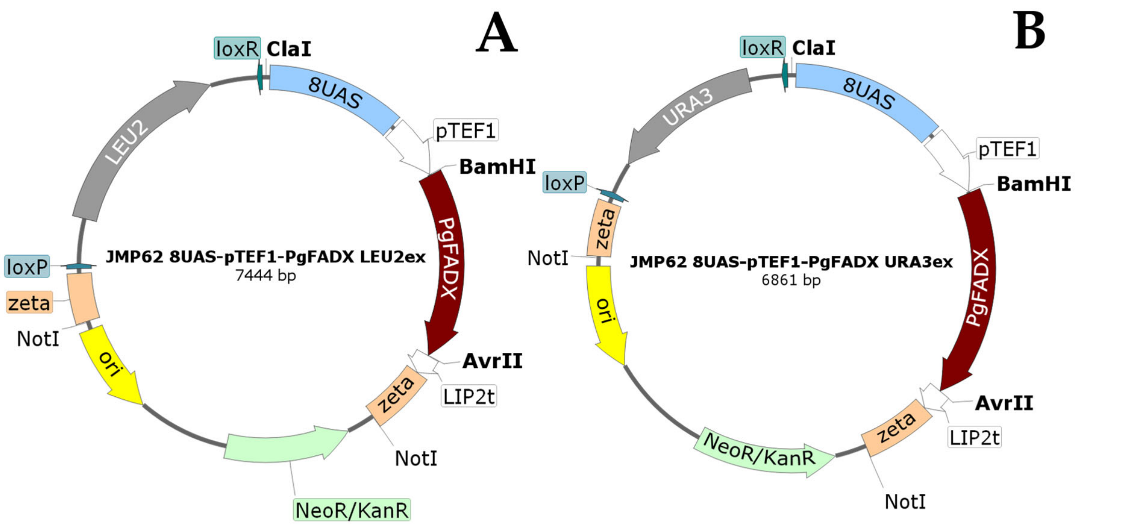

Supplementary Figure S1. Example of a plasmid gene map with the JMP62 base from *E. coli* strains EC78 (**A**) and E80 (**B**). The insertion cassette, prepared for transformation into *Y. lipolytica*, is delimited by sequences for the NotI restriction endonuclease. This cassette contains the *PgFADX* gene under the control of the constitutive 8UAS-pTEF1 promoter, flanked by restriction sites for ClaI and BamHI; the LIP2t terminator and a selection marker – the *LEU2* gene (**A**) or the *URA3* gene (**B**), bounded by loxR and loxP sequences, which serve to remove the selection marker from the *Y. lipolytica* genome using Cre recombinase if necessary to prepare an auxotrophic strain. The *PgFADX* gene was inserted into the cassette after the promoter sequence following cleavage with the restriction endonucleases BamHI and AvrII. The rest of the plasmid consists of the *NeoR/KanR* gene, which provides resistance to kanamycin, and the bacterial origin of replication *ori*.

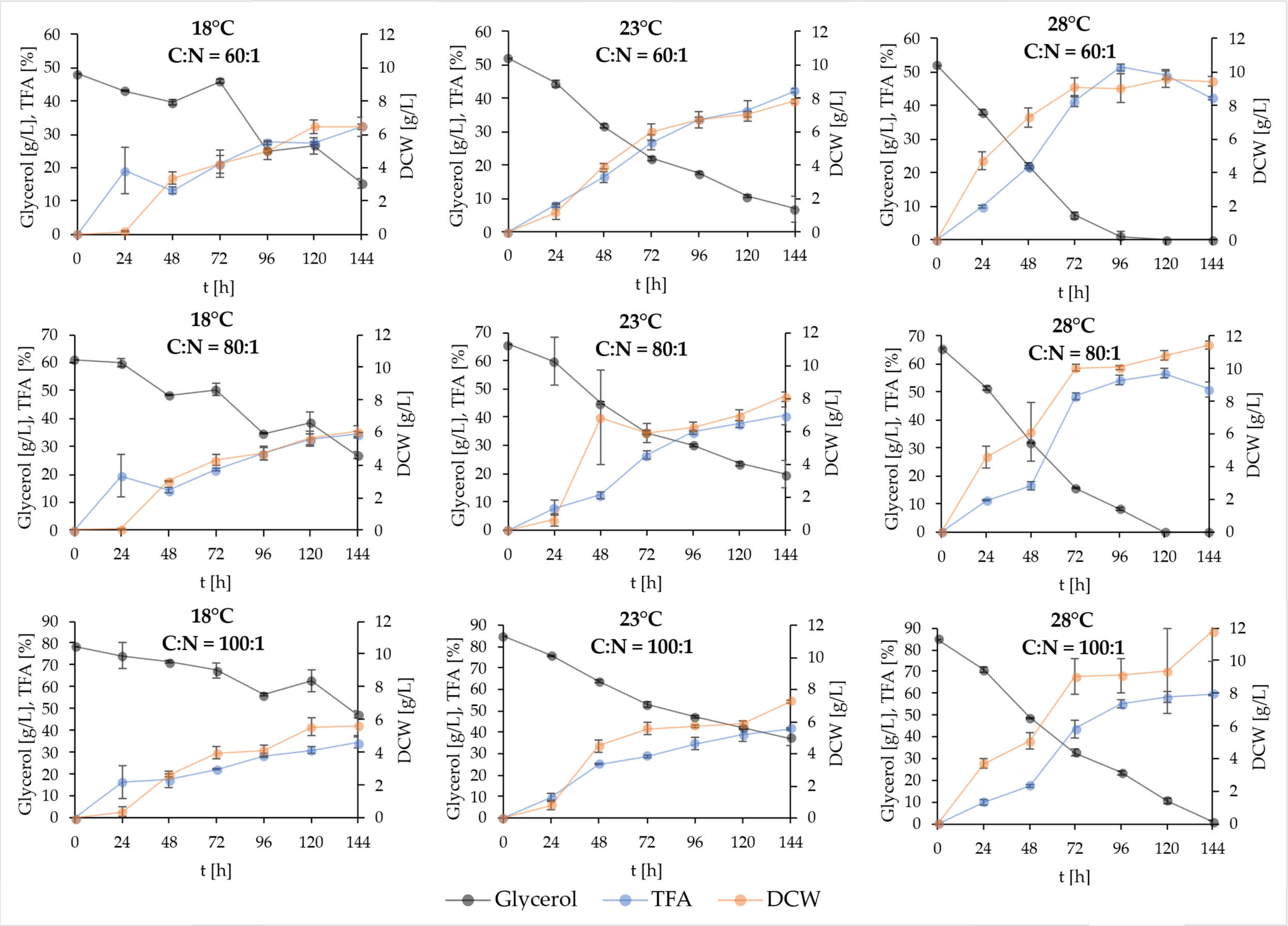

Supplementary Figure S2. Growth kinetics of *Y. lipolytica* YL129 strain in MedPGU medium. The effect of combining three different carbon-to-nitrogen ratios (C:N = 60:1; 80:1; 100:1) and three different cultivation temperatures (18, 23 and 28°C) were monitored. Abbreviations: total fatty acids (TFA), dry cell weight (DCW).

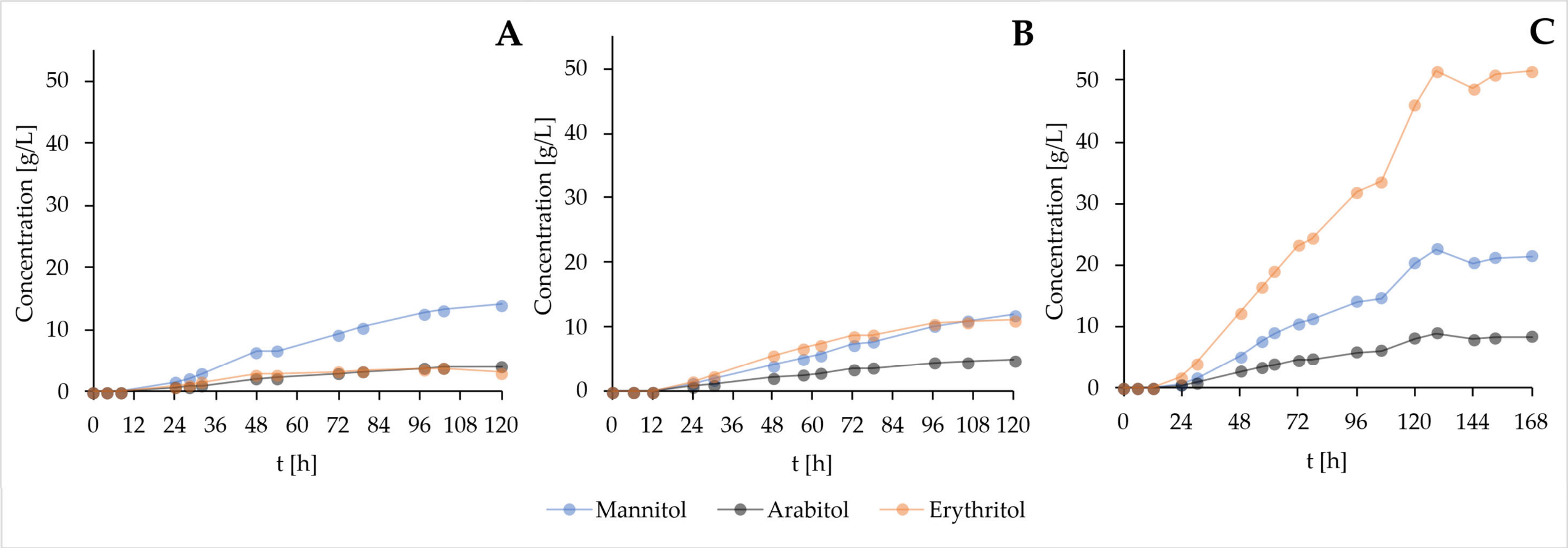

Supplementary Figure S3. Polyol production of *Y. lipolytica* YL129 strain for cultivation in a bioreactor in MedPGU medium (80 g/L pure glycerol; unadjusted pH) (**A**), in MedCGU medium (80 g/L crude glycerol; unadjusted pH) (**B**) and in MedCGU medium with double the amount of nitrogen and carbon (pH adjusted to 5.5 at the start of cultivation) (**C**). All fermentations were maintained and monitored until carbon depletion.

Supplementary Table S1. Composition of solutions for the preparation of MedPGU medium.

Solution	Component	Final concentration [g/L]	Stock solution
I. solution	Pure glycerol	60	none
	Yeast extract	1.5	
	KH_2PO_4	7	
	$Na_2HPO_4 \cdot 12H_2O$	5	
	$MnSO_4 \cdot H_2O$	$0.07 \cdot 10^{-3}$	
	$CuSO_4 \cdot 5H_2O$	$0.04 \cdot 10^{-3}$	
II. solution	$MgSO_4 \cdot 7H_2O$	1.5	50 x concentrated
III. solution	$Na_2EDTA \cdot 2H_2O$	$26.8 \cdot 10^{-3}$	50 x concentrated
	$FeSO_4 \cdot 7H_2O$	$20 \cdot 10^{-3}$	
IV. solution	$Na_2EDTA \cdot 2H_2O$	$10 \cdot 10^{-3}$	50 x concentrated
	$CaCl_2$	0.1	
	$ZnSO_4 \cdot 7H_2O$	$10 \cdot 10^{-3}$	

Supplementary Table S2. Official specifications of crude glycerol used in this study from the manufacturer (Mikrochem, Pezinok, Slovakia).

Glycerol content	min. 80.0 %
Ash	4.5-7.5 %
pH	5.0-7.5
Water	max. 9.0 %
Organic residue	0.5-2.0 %
Density	1.26-1.29 $g.cm^{-3}$
Appearance	golden brown to reddish brown liquid

Supplementary Table S3. Primers used in the study.

Name	Description	Sequence
PG9	LEU2in (forward)	TACGACGCATTGATGGAAGG
PG10	URA3in (forward)	TTGGTGGTGGTAACATCCAGAG
JH5	PgFADXin (reverse)	GTTGAAAGCGAGGTAAGAAGGC
VU7	PgDGAT1in (reverse)	GGCATGTTCGAGACCTGAC
VU8	PgDGAT2in (reverse)	GAGGGTCACGGGGAAGTATC
VU9	PgDGAT3in (reverse)	CCGGAACCACACCGAGC
VU10	PgPDATin (reverse)	CATCGGCGCAGTGGTG
